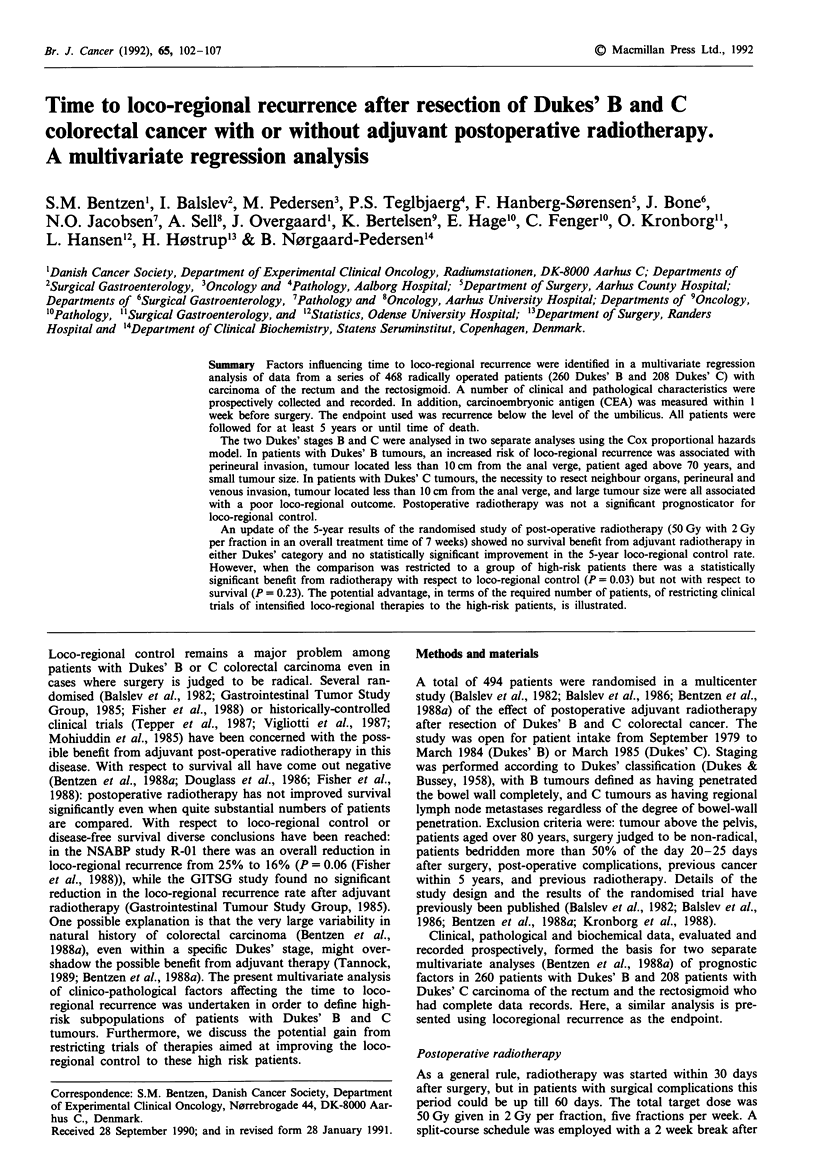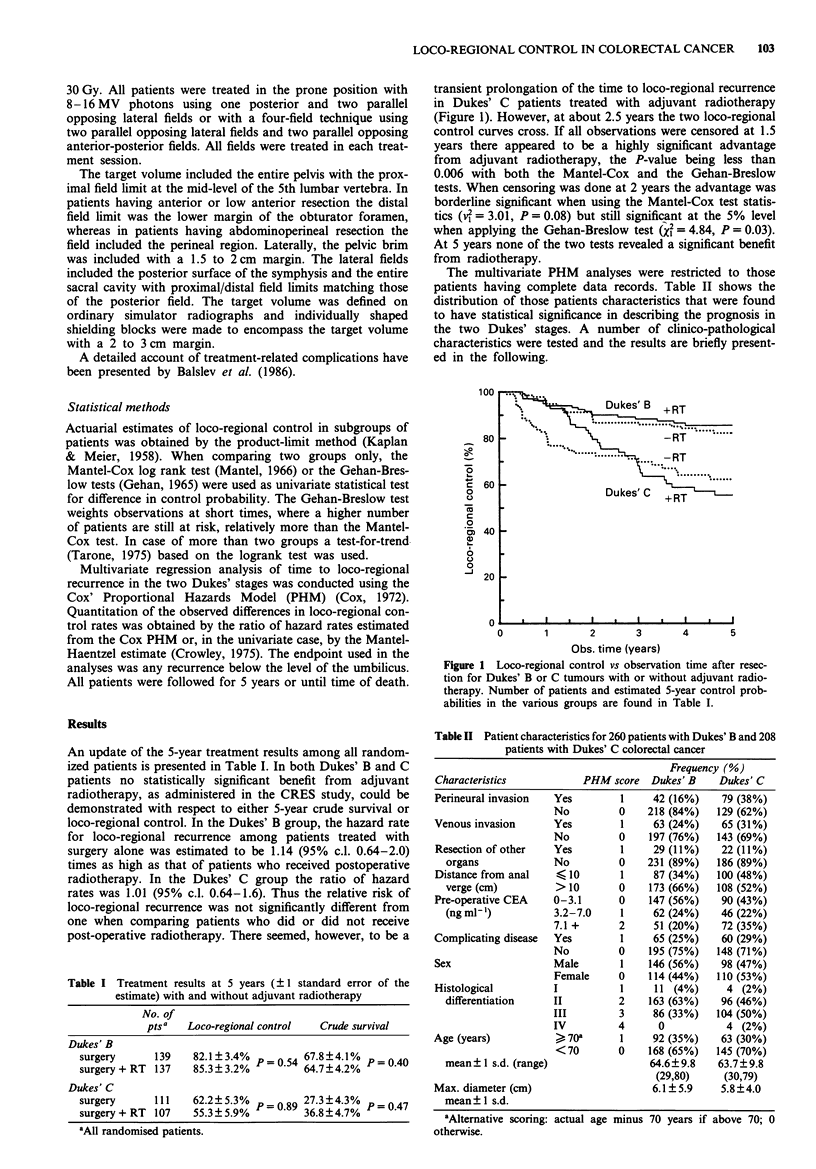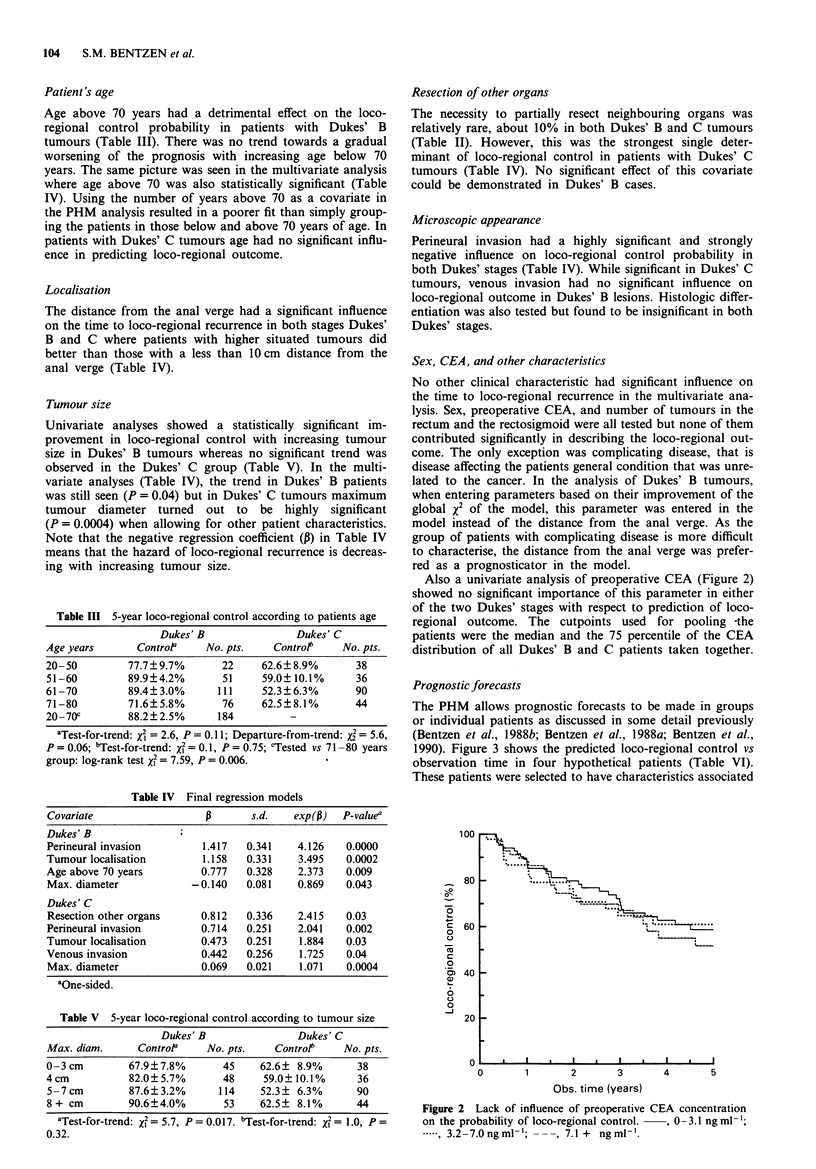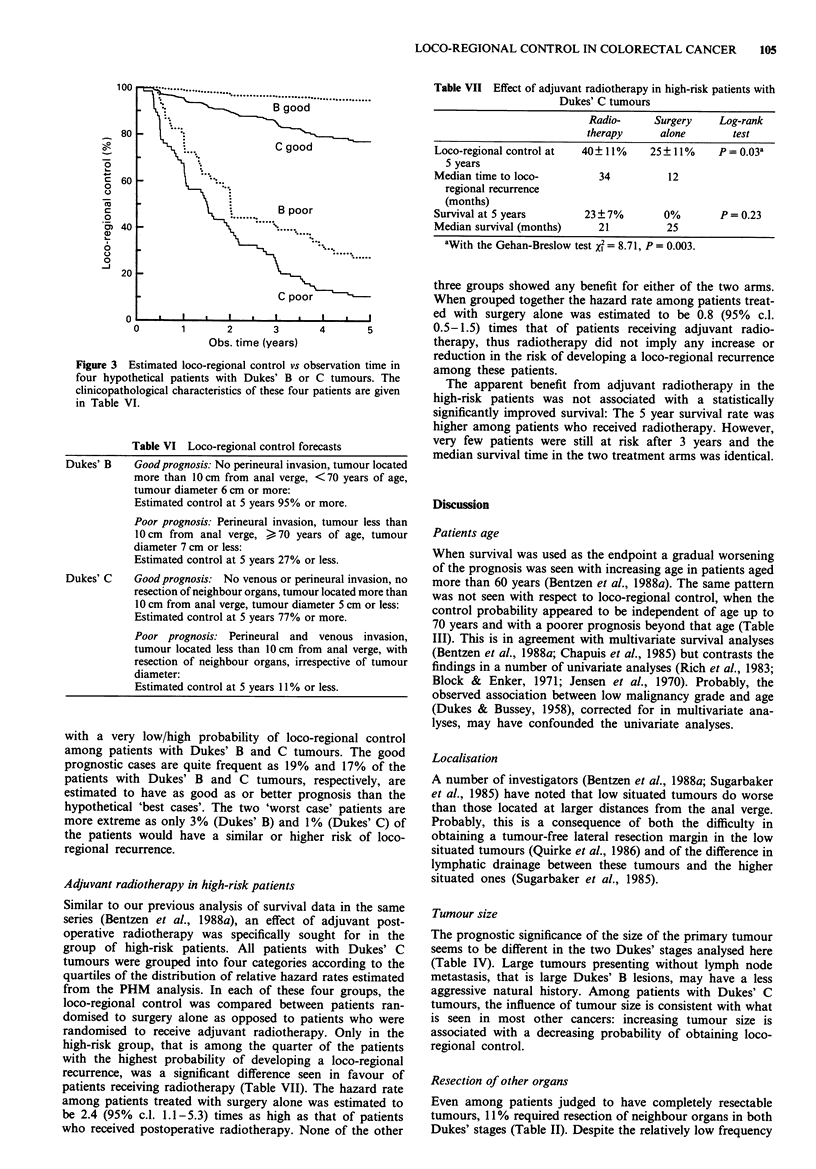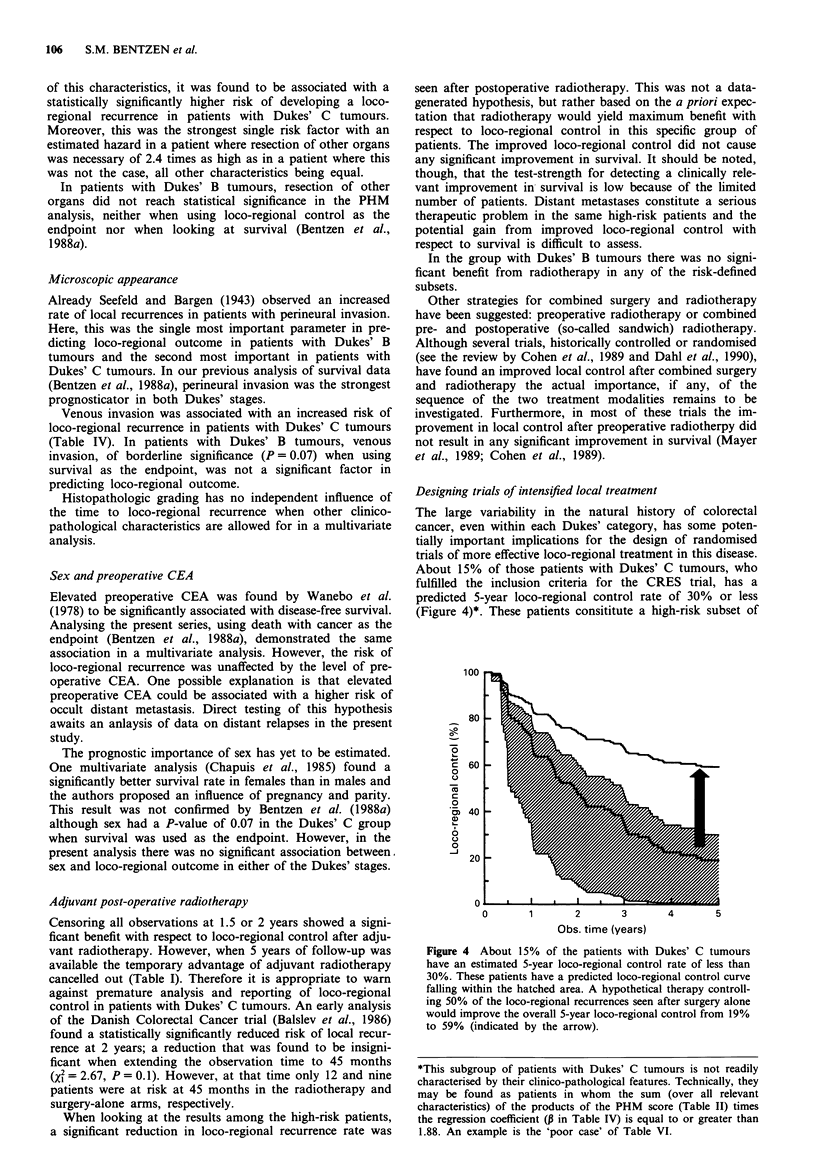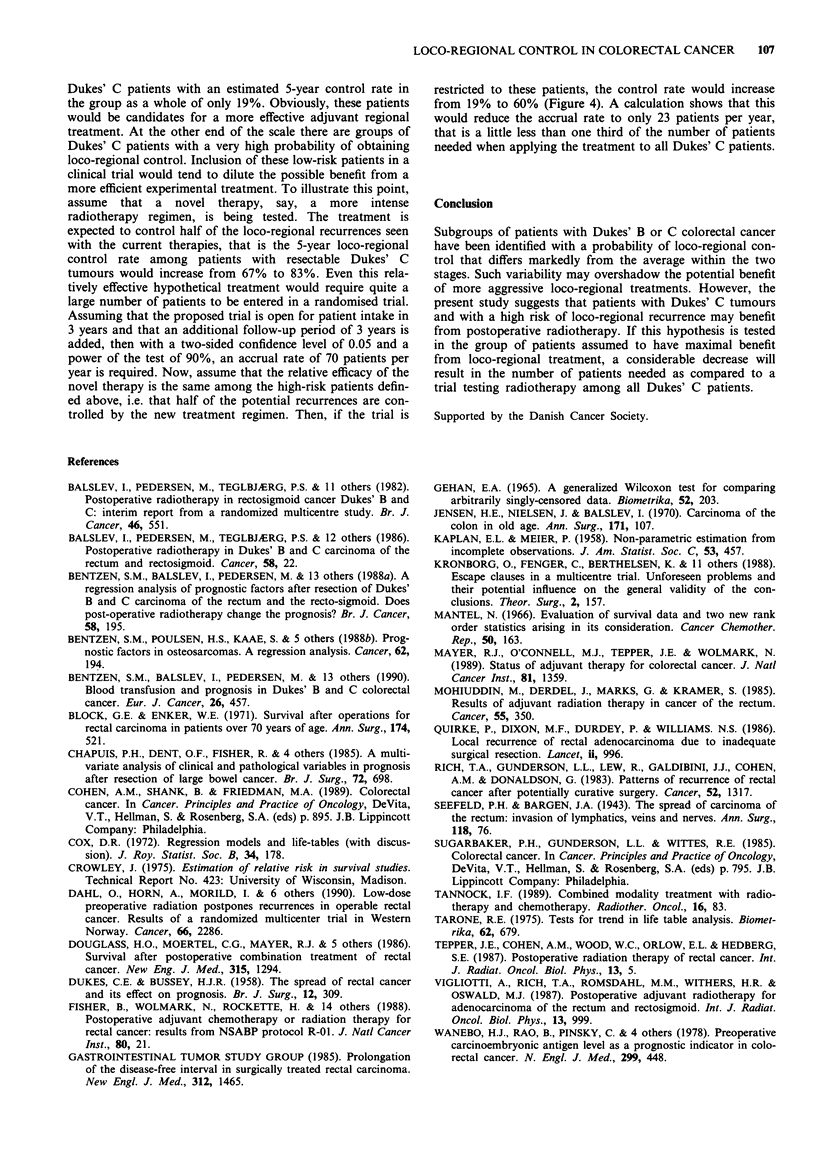# Time to loco-regional recurrence after resection of Dukes' B and C colorectal cancer with or without adjuvant postoperative radiotherapy. A multivariate regression analysis.

**DOI:** 10.1038/bjc.1992.19

**Published:** 1992-01

**Authors:** S. M. Bentzen, I. Balslev, M. Pedersen, P. S. Teglbjaerg, F. Hanberg-Sørensen, J. Bone, N. O. Jacobsen, A. Sell, J. Overgaard, K. Bertelsen

**Affiliations:** Danish Cancer Society, Department of Experimental Clinical Oncology, Aarhus C.

## Abstract

Factors influencing time to loco-regional recurrence were identified in a multivariate regression analysis of data from a series of 468 radically operated patients (260 Dukes' B and 208 Dukes' C) with carcinoma of the rectum and the rectosigmoid. A number of clinical and pathological characteristics were prospectively collected and recorded. In addition, carcinoembryonic antigen (CEA) was measured within 1 week before surgery. The endpoint used was recurrence below the level of the umbilicus. All patients were followed for at least 5 years or until time of death. The two Dukes' stages B and C were analysed in two separate analyses using the Cox proportional hazards model. In patients with Dukes' B tumours, an increased risk of loco-regional recurrence was associated with perineural invasion, tumour located less than 10 cm from the anal verge, patient aged above 70 years, and small tumour size. In patients with Dukes' C tumours, the necessity to resect neighbour organs, perineural and venous invasion, tumour located less than 10 cm from the anal verge, and large tumour size were all associated with a poor loco-regional outcome. Postoperative radiotherapy was not a significant prognosticator for loco-regional control. An update of the 5-year results of the randomised study of post-operative radiotherapy (50 Gy with 2 Gy per fraction in an overall treatment time of 7 weeks) showed no survival benefit from adjuvant radiotherapy in either Dukes' category and no statistically significant improvement in the 5-year loco-regional control rate. However, when the comparison was restricted to a group of high-risk patients there was a statistically significant benefit from radiotherapy with respect to loco-regional control (P = 0.03) but not with respect to survival (P = 0.23). The potential advantage, in terms of the required number of patients, of restricting clinical trials of intensified loco-regional therapies to the high-risk patients, is illustrated.


*Br. J. Cancer* (1992), **65**, 102–107 © Macmillan Press Ltd., 1992

### Time to loco-regional recurrence after resection of Dukes' B and C colorectal cancer with or without adjuvant postoperative radiotherapy. A multivariate regression analysis

S.M. Bentzen[1], I. Balslev[2], M. Pedersen[3], P.S. Teglbjaerg[4], F. Hanberg-Sørensen[5], J. Bone[6], N.O. Jacobsen[7], A. Sell[8], J. Overgaard[1], K. Bertelsen[9], E. Hage[10], C. Fenger[10], O. Kronborg[11], L. Hansen[12], H. Høstrup[13] & B. Nørgaard-Pedersen[14]

[1]*Danish Cancer Society, Department of Experimental Clinical Oncology, Radiumstationen, DK-8000 Aarhus C; Departments of* [2]*Surgical Gastroenterology,* [3]*Oncology and* [4]*Pathology, Aalborg Hospital;* [5]*Department of Surgery, Aarhus County Hospital; Departments of* [6]*Surgical Gastroenterology,* [7]*Pathology and* [8]*Oncology, Aarhus University Hospital; Departments of* [9]*Oncology,* [10]*Pathology,* [11]*Surgical Gastroenterology, and* [12]*Statistics, Odense University Hospital;* [13]*Department of Surgery, Randers Hospital and* [14]*Department of Clinical Biochemistry, Statens Seruminstitut, Copenhagen, Denmark.*

**Summary** Factors influencing time to loco-regional recurrence were identified in a multivariate regression analysis of data from a series of 468 radically operated patients (260 Dukes' B and 208 Dukes' C) with carcinoma of the rectum and the rectosigmoid. A number of clinical and pathological characteristics were prospectively collected and recorded. In addition, carcinoembryonic antigen (CEA) was measured within 1 week before surgery. The endpoint used was recurrence below the level of the umbilicus. All patients were followed for at least 5 years or until time of death.

The two Dukes' stages B and C were analysed in two separate analyses using the Cox proportional hazards model. In patients with Dukes' B tumours, an increased risk of loco-regional recurrence was associated with perineural invasion, tumour located less than 10 cm from the anal verge, patient aged above 70 years, and small tumour size. In patients with Dukes' C tumours, the necessity to resect neighbour organs, perineural and venous invasion, tumour located less than 10 cm from the anal verge, and large tumour size were all associated with a poor loco-regional outcome. Postoperative radiotherapy was not a significant prognosticator for loco-regional control.

An update of the 5-year results of the randomised study of post-operative radiotherapy (50 Gy with 2 Gy per fraction in an overall treatment time of 7 weeks) showed no survival benefit from adjuvant radiotherapy in either Dukes' category and no statistically significant improvement in the 5-year loco-regional control rate. However, when the comparison was restricted to a group of high-risk patients there was a statistically significant benefit from radiotherapy with respect to loco-regional control ($P = 0.03$) but not with respect to survival ($P = 0.23$). The potential advantage, in terms of the required number of patients, of restricting clinical trials of intensified loco-regional therapies to the high-risk patients, is illustrated.

Loco-regional control remains a major problem among patients with Dukes' B or C colorectal carcinoma even in cases where surgery is judged to be radical. Several randomised (Balslev *et al.*, 1982; Gastrointestinal Tumor Study Group, 1985; Fisher *et al.*, 1988) or historically-controlled clinical trials (Tepper *et al.*, 1987; Vigliotti *et al.*, 1987; Mohiuddin *et al.*, 1985) have been concerned with the possible benefit from adjuvant post-operative radiotherapy in this disease. With respect to survival all have come out negative (Bentzen *et al.*, 1988*a*; Douglass *et al.*, 1986; Fisher *et al.*, 1988): postoperative radiotherapy has not improved survival significantly even when quite substantial numbers of patients are compared. With respect to loco-regional control or disease-free survival diverse conclusions have been reached: in the NSABP study R-01 there was an overall reduction in loco-regional recurrence from 25% to 16% ($P = 0.06$ (Fisher *et al.*, 1988)), while the GITSG study found no significant reduction in the loco-regional recurrence rate after adjuvant radiotherapy (Gastrointestinal Tumour Study Group, 1985). One possible explanation is that the very large variability in natural history of colorectal carcinoma (Bentzen *et al.*, 1988*a*), even within a specific Dukes' stage, might overshadow the possible benefit from adjuvant therapy (Tannock, 1989; Bentzen *et al.*, 1988*a*). The present multivariate analysis of clinico-pathological factors affecting the time to loco-regional recurrence was undertaken in order to define high-risk subpopulations of patients with Dukes' B and C tumours. Furthermore, we discuss the potential gain from restricting trials of therapies aimed at improving the loco-regional control to these high risk patients.

Correspondence: S.M. Bentzen, Danish Cancer Society, Department of Experimental Clinical Oncology, Nørrebrogade 44, DK-8000 Aarhus C., Denmark.
Received 28 September 1990; and in revised form 28 January 1991.

### Methods and materials

A total of 494 patients were randomised in a multicenter study (Balslev *et al.*, 1982; Balslev *et al.*, 1986; Bentzen *et al.*, 1988*a*) of the effect of postoperative adjuvant radiotherapy after resection of Dukes' B and C colorectal cancer. The study was open for patient intake from September 1979 to March 1984 (Dukes' B) or March 1985 (Dukes' C). Staging was performed according to Dukes' classification (Dukes & Bussey, 1958), with B tumours defined as having penetrated the bowel wall completely, and C tumours as having regional lymph node metastases regardless of the degree of bowel-wall penetration. Exclusion criteria were: tumour above the pelvis, patients aged over 80 years, surgery judged to be non-radical, patients bedridden more than 50% of the day 20–25 days after surgery, post-operative complications, previous cancer within 5 years, and previous radiotherapy. Details of the study design and the results of the randomised trial have previously been published (Balslev *et al.*, 1982; Balslev *et al.*, 1986; Bentzen *et al.*, 1988*a*; Kronborg *et al.*, 1988).

Clinical, pathological and biochemical data, evaluated and recorded prospectively, formed the basis for two separate multivariate analyses (Bentzen *et al.*, 1988*a*) of prognostic factors in 260 patients with Dukes' B and 208 patients with Dukes' C carcinoma of the rectum and the rectosigmoid who had complete data records. Here, a similar analysis is presented using locoregional recurrence as the endpoint.

### *Postoperative radiotherapy*

As a general rule, radiotherapy was started within 30 days after surgery, but in patients with surgical complications this period could be up till 60 days. The total target dose was 50 Gy given in 2 Gy per fraction, five fractions per week. A split-course schedule was employed with a 2 week break after

30 Gy. All patients were treated in the prone position with 8–16 MV photons using one posterior and two parallel opposing lateral fields or with a four-field technique using two parallel opposing lateral fields and two parallel opposing anterior-posterior fields. All fields were treated in each treatment session.

The target volume included the entire pelvis with the proximal field limit at the mid-level of the 5th lumbar vertebra. In patients having anterior or low anterior resection the distal field limit was the lower margin of the obturator foramen, whereas in patients having abdominoperineal resection the field included the perineal region. Laterally, the pelvic brim was included with a 1.5 to 2 cm margin. The lateral fields included the posterior surface of the symphysis and the entire sacral cavity with proximal/distal field limits matching those of the posterior field. The target volume was defined on ordinary simulator radiographs and individually shaped shielding blocks were made to encompass the target volume with a 2 to 3 cm margin.

A detailed account of treatment-related complications have been presented by Balslev *et al.* (1986).

*Statistical methods*

Actuarial estimates of loco-regional control in subgroups of patients was obtained by the product-limit method (Kaplan & Meier, 1958). When comparing two groups only, the Mantel-Cox log rank test (Mantel, 1966) or the Gehan-Breslow tests (Gehan, 1965) were used as univariate statistical test for difference in control probability. The Gehan-Breslow test weights observations at short times, where a higher number of patients are still at risk, relatively more than the Mantel-Cox test. In case of more than two groups a test-for-trend (Tarone, 1975) based on the logrank test was used.

Multivariate regression analysis of time to loco-regional recurrence in the two Dukes' stages was conducted using the Cox' Proportional Hazards Model (PHM) (Cox, 1972). Quantitation of the observed differences in loco-regional control rates was obtained by the ratio of hazard rates estimated from the Cox PHM or, in the univariate case, by the Mantel-Haentzel estimate (Crowley, 1975). The endpoint used in the analyses was any recurrence below the level of the umbilicus. All patients were followed for 5 years or until time of death.

### Results

An update of the 5-year treatment results among all randomized patients is presented in Table I. In both Dukes' B and C patients no statistically significant benefit from adjuvant radiotherapy, as administered in the CRES study, could be demonstrated with respect to either 5-year crude survival or loco-regional control. In the Dukes' B group, the hazard rate for loco-regional recurrence among patients treated with surgery alone was estimated to be 1.14 (95% c.l. 0.64–2.0) times as high as that of patients who received postoperative radiotherapy. In the Dukes' C group the ratio of hazard rates was 1.01 (95% c.l. 0.64–1.6). Thus the relative risk of loco-regional recurrence was not significantly different from one when comparing patients who did or did not receive post-operative radiotherapy. There seemed, however, to be a

**Table I** Treatment results at 5 years (±1 standard error of the estimate) with and without adjuvant radiotherapy

| | No. of pts[a] | Loco-regional control | | Crude survival | |
|---|---|---|---|---|---|
| *Dukes' B* | | | | | |
| surgery | 139 | 82.1±3.4% | $P=0.54$ | 67.8±4.1% | $P=0.40$ |
| surgery + RT | 137 | 85.3±3.2% | | 64.7±4.2% | |
| *Dukes' C* | | | | | |
| surgery | 111 | 62.2±5.3% | $P=0.89$ | 27.3±4.3% | $P=0.47$ |
| surgery + RT | 107 | 55.3±5.9% | | 36.8±4.7% | |

[a]All randomised patients.

transient prolongation of the time to loco-regional recurrence in Dukes' C patients treated with adjuvant radiotherapy (Figure 1). However, at about 2.5 years the two loco-regional control curves cross. If all observations were censored at 1.5 years there appeared to be a highly significant advantage from adjuvant radiotherapy, the *P*-value being less than 0.006 with both the Mantel-Cox and the Gehan-Breslow tests. When censoring was done at 2 years the advantage was borderline significant when using the Mantel-Cox test statistics ($\nu_1^2=3.01$, $P=0.08$) but still significant at the 5% level when applying the Gehan-Breslow test ($\chi_1^2=4.84$, $P=0.03$). At 5 years none of the two tests revealed a significant benefit from radiotherapy.

The multivariate PHM analyses were restricted to those patients having complete data records. Table II shows the distribution of those patients characteristics that were found to have statistical significance in describing the prognosis in the two Dukes' stages. A number of clinico-pathological characteristics were tested and the results are briefly presented in the following.

**Figure 1** Loco-regional control *vs* observation time after resection for Dukes' B or C tumours with or without adjuvant radiotherapy. Number of patients and estimated 5-year control probabilities in the various groups are found in Table I.

**Table II** Patient characteristics for 260 patients with Dukes' B and 208 patients with Dukes' C colorectal cancer

| Characteristics | | PHM score | Frequency (%) Dukes' B | Dukes' C |
|---|---|---|---|---|
| Perineural invasion | Yes | 1 | 42 (16%) | 79 (38%) |
| | No | 0 | 218 (84%) | 129 (62%) |
| Venous invasion | Yes | 1 | 63 (24%) | 65 (31%) |
| | No | 0 | 197 (76%) | 143 (69%) |
| Resection of other organs | Yes | 1 | 29 (11%) | 22 (11%) |
| | No | 0 | 231 (89%) | 186 (89%) |
| Distance from anal verge (cm) | ⩽10 | 1 | 87 (34%) | 100 (48%) |
| | >10 | 0 | 173 (66%) | 108 (52%) |
| Pre-operative CEA ($ng\ ml^{-1}$) | 0–3.1 | 0 | 147 (56%) | 90 (43%) |
| | 3.2–7.0 | 1 | 62 (24%) | 46 (22%) |
| | 7.1 + | 2 | 51 (20%) | 72 (35%) |
| Complicating disease | Yes | 1 | 65 (25%) | 60 (29%) |
| | No | 0 | 195 (75%) | 148 (71%) |
| Sex | Male | 1 | 146 (56%) | 98 (47%) |
| | Female | 0 | 114 (44%) | 110 (53%) |
| Histological differentiation | I | 1 | 11 (4%) | 4 (2%) |
| | II | 2 | 163 (63%) | 96 (46%) |
| | III | 3 | 86 (33%) | 104 (50%) |
| | IV | 4 | 0 | 4 (2%) |
| Age (years) | ⩾70[a] | 1 | 92 (35%) | 63 (30%) |
| | <70 | 0 | 168 (65%) | 145 (70%) |
| mean±1 s.d. (range) | | | 64.6±9.8 (29,80) | 63.7±9.8 (30,79) |
| Max. diameter (cm) mean±1 s.d. | | | 6.1±5.9 | 5.8±4.0 |

[a]Alternative scoring: actual age minus 70 years if above 70; 0 otherwise.

*Patient's age*

Age above 70 years had a detrimental effect on the loco-regional control probability in patients with Dukes' B tumours (Table III). There was no trend towards a gradual worsening of the prognosis with increasing age below 70 years. The same picture was seen in the multivariate analysis where age above 70 was also statistically significant (Table IV). Using the number of years above 70 as a covariate in the PHM analysis resulted in a poorer fit than simply grouping the patients in those below and above 70 years of age. In patients with Dukes' C tumours age had no significant influence in predicting loco-regional outcome.

*Localisation*

The distance from the anal verge had a significant influence on the time to loco-regional recurrence in both stages Dukes' B and C where patients with higher situated tumours did better than those with a less than 10 cm distance from the anal verge (Table IV).

*Tumour size*

Univariate analyses showed a statistically significant improvement in loco-regional control with increasing tumour size in Dukes' B tumours whereas no significant trend was observed in the Dukes' C group (Table V). In the multivariate analyses (Table IV), the trend in Dukes' B patients was still seen ($P = 0.04$) but in Dukes' C tumours maximum tumour diameter turned out to be highly significant ($P = 0.0004$) when allowing for other patient characteristics. Note that the negative regression coefficient ($\beta$) in Table IV means that the hazard of loco-regional recurrence is decreasing with increasing tumour size.

**Table III** 5-year loco-regional control according to patients age

| Age years | Dukes' B Control[a] | Dukes' B No. pts. | Dukes' C Control[b] | Dukes' C No. pts. |
|---|---|---|---|---|
| 20–50 | 77.7±9.7% | 22 | 62.6±8.9% | 38 |
| 51–60 | 89.9±4.2% | 51 | 59.0±10.1% | 36 |
| 61–70 | 89.4±3.0% | 111 | 52.3±6.3% | 90 |
| 71–80 | 71.6±5.8% | 76 | 62.5±8.1% | 44 |
| 20–70[c] | 88.2±2.5% | 184 | – | |

[a]Test-for-trend: $\chi_1^2 = 2.6$, $P = 0.11$; Departure-from-trend: $\chi_2^2 = 5.6$, $P = 0.06$; [b]Test-for-trend: $\chi_1^2 = 0.1$, $P = 0.75$; [c]Tested *vs* 71–80 years group: log-rank test $\chi_1^2 = 7.59$, $P = 0.006$.

**Table IV** Final regression models

| Covariate | $\beta$ | s.d. | exp($\beta$) | P-value[a] |
|---|---|---|---|---|
| *Dukes' B* | | | | |
| Perineural invasion | 1.417 | 0.341 | 4.126 | 0.0000 |
| Tumour localisation | 1.158 | 0.331 | 3.495 | 0.0002 |
| Age above 70 years | 0.777 | 0.328 | 2.373 | 0.009 |
| Max. diameter | −0.140 | 0.081 | 0.869 | 0.043 |
| *Dukes' C* | | | | |
| Resection other organs | 0.812 | 0.336 | 2.415 | 0.03 |
| Perineural invasion | 0.714 | 0.251 | 2.041 | 0.002 |
| Tumour localisation | 0.473 | 0.251 | 1.884 | 0.03 |
| Venous invasion | 0.442 | 0.256 | 1.725 | 0.04 |
| Max. diameter | 0.069 | 0.021 | 1.071 | 0.0004 |

[a]One-sided.

**Table V** 5-year loco-regional control according to tumour size

| Max. diam. | Dukes' B Control[a] | Dukes' B No. pts. | Dukes' C Control[b] | Dukes' C No. pts. |
|---|---|---|---|---|
| 0–3 cm | 67.9±7.8% | 45 | 62.6± 8.9% | 38 |
| 4 cm | 82.0±5.7% | 48 | 59.0±10.1% | 36 |
| 5–7 cm | 87.6±3.2% | 114 | 52.3± 6.3% | 90 |
| 8+ cm | 90.6±4.0% | 53 | 62.5± 8.1% | 44 |

[a]Test-for-trend: $\chi_1^2 = 5.7$, $P = 0.017$. [b]Test-for-trend: $\chi_1^2 = 1.0$, $P = 0.32$.

*Resection of other organs*

The necessity to partially resect neighbouring organs was relatively rare, about 10% in both Dukes' B and C tumours (Table II). However, this was the strongest single determinant of loco-regional control in patients with Dukes' C tumours (Table IV). No significant effect of this covariate could be demonstrated in Dukes' B cases.

*Microscopic appearance*

Perineural invasion had a highly significant and strongly negative influence on loco-regional control probability in both Dukes' stages (Table IV). While significant in Dukes' C tumours, venous invasion had no significant influence on loco-regional outcome in Dukes' B lesions. Histologic differentiation was also tested but found to be insignificant in both Dukes' stages.

*Sex, CEA, and other characteristics*

No other clinical characteristic had significant influence on the time to loco-regional recurrence in the multivariate analysis. Sex, preoperative CEA, and number of tumours in the rectum and the rectosigmoid were all tested but none of them contributed significantly in describing the loco-regional outcome. The only exception was complicating disease, that is disease affecting the patients general condition that was unrelated to the cancer. In the analysis of Dukes' B tumours, when entering parameters based on their improvement of the global $\chi^2$ of the model, this parameter was entered in the model instead of the distance from the anal verge. As the group of patients with complicating disease is more difficult to characterise, the distance from the anal verge was preferred as a prognosticator in the model.

Also a univariate analysis of preoperative CEA (Figure 2) showed no significant importance of this parameter in either of the two Dukes' stages with respect to prediction of loco-regional outcome. The cutpoints used for pooling the patients were the median and the 75 percentile of the CEA distribution of all Dukes' B and C patients taken together.

*Prognostic forecasts*

The PHM allows prognostic forecasts to be made in groups or individual patients as discussed in some detail previously (Bentzen *et al.*, 1988*b*; Bentzen *et al.*, 1988*a*; Bentzen *et al.*, 1990). Figure 3 shows the predicted loco-regional control *vs* observation time in four hypothetical patients (Table VI). These patients were selected to have characteristics associated

**Figure 2** Lack of influence of preoperative CEA concentration on the probability of loco-regional control. ——, 0–3.1 ng ml$^{-1}$; ·····, 3.2–7.0 ng ml$^{-1}$; – – –, 7.1 + ng ml$^{-1}$.

**Figure 3** Estimated loco-regional control *vs* observation time in four hypothetical patients with Dukes' B or C tumours. The clinicopathological characteristics of these four patients are given in Table VI.

**Table VI** Loco-regional control forecasts

| | |
|---|---|
| Dukes' B | *Good prognosis:* No perineural invasion, tumour located more than 10 cm from anal verge, <70 years of age, tumour diameter 6 cm or more: Estimated control at 5 years 95% or more. |
| | *Poor prognosis:* Perineural invasion, tumour less than 10 cm from anal verge, ⩾70 years of age, tumour diameter 7 cm or less: Estimated control at 5 years 27% or less. |
| Dukes' C | *Good prognosis:* No venous or perineural invasion, no resection of neighbour organs, tumour located more than 10 cm from anal verge, tumour diameter 5 cm or less: Estimated control at 5 years 77% or more. |
| | *Poor prognosis:* Perineural and venous invasion, tumour located less than 10 cm from anal verge, with resection of neighbour organs, irrespective of tumour diameter: Estimated control at 5 years 11% or less. |

with a very low/high probability of loco-regional control among patients with Dukes' B and C tumours. The good prognostic cases are quite frequent as 19% and 17% of the patients with Dukes' B and C tumours, respectively, are estimated to have as good as or better prognosis than the hypothetical 'best cases'. The two 'worst case' patients are more extreme as only 3% (Dukes' B) and 1% (Dukes' C) of the patients would have a similar or higher risk of loco-regional recurrence.

### *Adjuvant radiotherapy in high-risk patients*

Similar to our previous analysis of survival data in the same series (Bentzen *et al.*, 1988*a*), an effect of adjuvant post-operative radiotherapy was specifically sought for in the group of high-risk patients. All patients with Dukes' C tumours were grouped into four categories according to the quartiles of the distribution of relative hazard rates estimated from the PHM analysis. In each of these four groups, the loco-regional control was compared between patients randomised to surgery alone as opposed to patients who were randomised to receive adjuvant radiotherapy. Only in the high-risk group, that is among the quarter of the patients with the highest probability of developing a loco-regional recurrence, was a significant difference seen in favour of patients receiving radiotherapy (Table VII). The hazard rate among patients treated with surgery alone was estimated to be 2.4 (95% c.l. 1.1–5.3) times as high as that of patients who received postoperative radiotherapy. None of the other

**Table VII** Effect of adjuvant radiotherapy in high-risk patients with Dukes' C tumours

| | *Radio-therapy* | *Surgery alone* | *Log-rank test* |
|---|---|---|---|
| Loco-regional control at 5 years | 40±11% | 25±11% | $P = 0.03^a$ |
| Median time to loco-regional recurrence (months) | 34 | 12 | |
| Survival at 5 years | 23±7% | 0% | $P = 0.23$ |
| Median survival (months) | 21 | 25 | |

[a]With the Gehan-Breslow test $\chi_1^2 = 8.71$, $P = 0.003$.

three groups showed any benefit for either of the two arms. When grouped together the hazard rate among patients treated with surgery alone was estimated to be 0.8 (95% c.l. 0.5–1.5) times that of patients receiving adjuvant radiotherapy, thus radiotherapy did not imply any increase or reduction in the risk of developing a loco-regional recurrence among these patients.

The apparent benefit from adjuvant radiotherapy in the high-risk patients was not associated with a statistically significantly improved survival: The 5 year survival rate was higher among patients who received radiotherapy. However, very few patients were still at risk after 3 years and the median survival time in the two treatment arms was identical.

### Discussion

### *Patients age*

When survival was used as the endpoint a gradual worsening of the prognosis was seen with increasing age in patients aged more than 60 years (Bentzen *et al.*, 1988*a*). The same pattern was not seen with respect to loco-regional control, when the control probability appeared to be independent of age up to 70 years and with a poorer prognosis beyond that age (Table III). This is in agreement with multivariate survival analyses (Bentzen *et al.*, 1988*a*; Chapuis *et al.*, 1985) but contrasts the findings in a number of univariate analyses (Rich *et al.*, 1983; Block & Enker, 1971; Jensen *et al.*, 1970). Probably, the observed association between low malignancy grade and age (Dukes & Bussey, 1958), corrected for in multivariate analyses, may have confounded the univariate analyses.

### *Localisation*

A number of investigators (Bentzen *et al.*, 1988*a*; Sugarbaker *et al.*, 1985) have noted that low situated tumours do worse than those located at larger distances from the anal verge. Probably, this is a consequence of both the difficulty in obtaining a tumour-free lateral resection margin in the low situated tumours (Quirke *et al.*, 1986) and of the difference in lymphatic drainage between these tumours and the higher situated ones (Sugarbaker *et al.*, 1985).

### *Tumour size*

The prognostic significance of the size of the primary tumour seems to be different in the two Dukes' stages analysed here (Table IV). Large tumours presenting without lymph node metastasis, that is large Dukes' B lesions, may have a less aggressive natural history. Among patients with Dukes' C tumours, the influence of tumour size is consistent with what is seen in most other cancers: increasing tumour size is associated with a decreasing probability of obtaining loco-regional control.

### *Resection of other organs*

Even among patients judged to have completely resectable tumours, 11% required resection of neighbour organs in both Dukes' stages (Table II). Despite the relatively low frequency of this characteristics, it was found to be associated with a statistically significantly higher risk of developing a loco-regional recurrence in patients with Dukes' C tumours. Moreover, this was the strongest single risk factor with an estimated hazard in a patient where resection of other organs was necessary of 2.4 times as high as in a patient where this was not the case, all other characteristics being equal.

In patients with Dukes' B tumours, resection of other organs did not reach statistical significance in the PHM analysis, neither when using loco-regional control as the endpoint nor when looking at survival (Bentzen *et al.*, 1988*a*).

*Microscopic appearance*

Already Seefeld and Bargen (1943) observed an increased rate of local recurrences in patients with perineural invasion. Here, this was the single most important parameter in predicting loco-regional outcome in patients with Dukes' B tumours and the second most important in patients with Dukes' C tumours. In our previous analysis of survival data (Bentzen *et al.*, 1988*a*), perineural invasion was the strongest prognosticator in both Dukes' stages.

Venous invasion was associated with an increased risk of loco-regional recurrence in patients with Dukes' C tumours (Table IV). In patients with Dukes' B tumours, venous invasion, of borderline significance ($P = 0.07$) when using survival as the endpoint, was not a significant factor in predicting loco-regional outcome.

Histopathologic grading has no independent influence of the time to loco-regional recurrence when other clinico-pathological characteristics are allowed for in a multivariate analysis.

*Sex and preoperative CEA*

Elevated preoperative CEA was found by Wanebo *et al.* (1978) to be significantly associated with disease-free survival. Analysing the present series, using death with cancer as the endpoint (Bentzen *et al.*, 1988*a*), demonstrated the same association in a multivariate analysis. However, the risk of loco-regional recurrence was unaffected by the level of preoperative CEA. One possible explanation is that elevated preoperative CEA could be associated with a higher risk of occult distant metastasis. Direct testing of this hypothesis awaits an anlaysis of data on distant relapses in the present study.

The prognostic importance of sex has yet to be estimated. One multivariate analysis (Chapuis *et al.*, 1985) found a significantly better survival rate in females than in males and the authors proposed an influence of pregnancy and parity. This result was not confirmed by Bentzen *et al.* (1988*a*) although sex had a *P*-value of 0.07 in the Dukes' C group when survival was used as the endpoint. However, in the present analysis there was no significant association between sex and loco-regional outcome in either of the Dukes' stages.

*Adjuvant post-operative radiotherapy*

Censoring all observations at 1.5 or 2 years showed a significant benefit with respect to loco-regional control after adjuvant radiotherapy. However, when 5 years of follow-up was available the temporary advantage of adjuvant radiotherapy cancelled out (Table I). Therefore it is appropriate to warn against premature analysis and reporting of loco-regional control in patients with Dukes' C tumours. An early analysis of the Danish Colorectal Cancer trial (Balslev *et al.*, 1986) found a statistically significantly reduced risk of local recurrence at 2 years; a reduction that was found to be insignificant when extending the observation time to 45 months ($\chi^2_1 = 2.67$, $P = 0.1$). However, at that time only 12 and nine patients were at risk at 45 months in the radiotherapy and surgery-alone arms, respectively.

When looking at the results among the high-risk patients, a significant reduction in loco-regional recurrence rate was seen after postoperative radiotherapy. This was not a data-generated hypothesis, but rather based on the *a priori* expectation that radiotherapy would yield maximum benefit with respect to loco-regional control in this specific group of patients. The improved loco-regional control did not cause any significant improvement in survival. It should be noted, though, that the test-strength for detecting a clinically relevant improvement in survival is low because of the limited number of patients. Distant metastases constitute a serious therapeutic problem in the same high-risk patients and the potential gain from improved loco-regional control with respect to survival is difficult to assess.

In the group with Dukes' B tumours there was no significant benefit from radiotherapy in any of the risk-defined subsets.

Other strategies for combined surgery and radiotherapy have been suggested: preoperative radiotherapy or combined pre- and postoperative (so-called sandwich) radiotherapy. Although several trials, historically controlled or randomised (see the review by Cohen *et al.*, 1989 and Dahl *et al.*, 1990), have found an improved local control after combined surgery and radiotherapy the actual importance, if any, of the sequence of the two treatment modalities remains to be investigated. Furthermore, in most of these trials the improvement in local control after preoperative radiotherpy did not result in any significant improvement in survival (Mayer *et al.*, 1989; Cohen *et al.*, 1989).

*Designing trials of intensified local treatment*

The large variability in the natural history of colorectal cancer, even within each Dukes' category, has some potentially important implications for the design of randomised trials of more effective loco-regional treatment in this disease. About 15% of those patients with Dukes' C tumours, who fulfilled the inclusion criteria for the CRES trial, has a predicted 5-year loco-regional control rate of 30% or less (Figure 4)*. These patients consititute a high-risk subset of

**Figure 4** About 15% of the patients with Dukes' C tumours have an estimated 5-year loco-regional control rate of less than 30%. These patients have a predicted loco-regional control curve falling within the hatched area. A hypothetical therapy controlling 50% of the loco-regional recurrences seen after surgery alone would improve the overall 5-year loco-regional control from 19% to 59% (indicated by the arrow).

*This subgroup of patients with Dukes' C tumours is not readily characterised by their clinico-pathological features. Technically, they may be found as patients in whom the sum (over all relevant characteristics) of the products of the PHM score (Table II) times the regression coefficient ($\beta$ in Table IV) is equal to or greater than 1.88. An example is the 'poor case' of Table VI.

Dukes' C patients with an estimated 5-year control rate in the group as a whole of only 19%. Obviously, these patients would be candidates for a more effective adjuvant regional treatment. At the other end of the scale there are groups of Dukes' C patients with a very high probability of obtaining loco-regional control. Inclusion of these low-risk patients in a clinical trial would tend to dilute the possible benefit from a more efficient experimental treatment. To illustrate this point, assume that a novel therapy, say, a more intense radiotherapy regimen, is being tested. The treatment is expected to control half of the loco-regional recurrences seen with the current therapies, that is the 5-year loco-regional control rate among patients with resectable Dukes' C tumours would increase from 67% to 83%. Even this relatively effective hypothetical treatment would require quite a large number of patients to be entered in a randomised trial. Assuming that the proposed trial is open for patient intake in 3 years and that an additional follow-up period of 3 years is added, then with a two-sided confidence level of 0.05 and a power of the test of 90%, an accrual rate of 70 patients per year is required. Now, assume that the relative efficacy of the novel therapy is the same among the high-risk patients defined above, i.e. that half of the potential recurrences are controlled by the new treatment regimen. Then, if the trial is restricted to these patients, the control rate would increase from 19% to 60% (Figure 4). A calculation shows that this would reduce the accrual rate to only 23 patients per year, that is a little less than one third of the number of patients needed when applying the treatment to all Dukes' C patients.

### Conclusion

Subgroups of patients with Dukes' B or C colorectal cancer have been identified with a probability of loco-regional control that differs markedly from the average within the two stages. Such variability may overshadow the potential benefit of more aggressive loco-regional treatments. However, the present study suggests that patients with Dukes' C tumours and with a high risk of loco-regional recurrence may benefit from postoperative radiotherapy. If this hypothesis is tested in the group of patients assumed to have maximal benefit from loco-regional treatment, a considerable decrease will result in the number of patients needed as compared to a trial testing radiotherapy among all Dukes' C patients.

Supported by the Danish Cancer Society.

### References

BALSLEV, I., PEDERSEN, M., TEGLBJÆRG, P.S. & 11 others (1982). Postoperative radiotherapy in rectosigmoid cancer Dukes' B and C: interim report from a randomized multicentre study. *Br. J. Cancer*, **46**, 551.

BALSLEV, I., PEDERSEN, M., TEGLBJÆRG, P.S. & 12 others (1986). Postoperative radiotherapy in Dukes' B and C carcinoma of the rectum and rectosigmoid. *Cancer*, **58**, 22.

BENTZEN, S.M., BALSLEV, I., PEDERSEN, M. & 13 others (1988*a*). A regression analysis of prognostic factors after resection of Dukes' B and C carcinoma of the rectum and the recto-sigmoid. Does post-operative radiotherapy change the prognosis? *Br. J. Cancer*, **58**, 195.

BENTZEN, S.M., POULSEN, H.S., KAAE, S. & 5 others (1988*b*). Prognostic factors in osteosarcomas. A regression analysis. *Cancer*, **62**, 194.

BENTZEN, S.M., BALSLEV, I., PEDERSEN, M. & 13 others (1990). Blood transfusion and prognosis in Dukes' B and C colorectal cancer. *Eur. J. Cancer*, **26**, 457.

BLOCK, G.E. & ENKER, W.E. (1971). Survival after operations for rectal carcinoma in patients over 70 years of age. *Ann. Surg.*, **174**, 521.

CHAPUIS, P.H., DENT, O.F., FISHER, R. & 4 others (1985). A multivariate analysis of clinical and pathological variables in prognosis after resection of large bowel cancer. *Br. J. Surg.*, **72**, 698.

COHEN, A.M., SHANK, B. & FRIEDMAN, M.A. (1989). Colorectal cancer. In *Cancer. Principles and Practice of Oncology*, DeVita, V.T., Hellman, S. & Rosenberg, S.A. (eds) p. 895. J.B. Lippincott Company: Philadelphia.

COX, D.R. (1972). Regression models and life-tables (with discussion). *J. Roy. Statist. Soc. B*, **34**, 178.

CROWLEY, J. (1975). *Estimation of relative risk in survival studies.* Technical Report No. 423: University of Wisconsin, Madison.

DAHL, O., HORN, A., MORILD, I. & 6 others (1990). Low-dose preoperative radiation postpones recurrences in operable rectal cancer. Results of a randomized multicenter trial in Western Norway. *Cancer*, **66**, 2286.

DOUGLASS, H.O., MOERTEL, C.G., MAYER, R.J. & 5 others (1986). Survival after postoperative combination treatment of rectal cancer. *New Eng. J. Med.*, **315**, 1294.

DUKES, C.E. & BUSSEY, H.J.R. (1958). The spread of rectal cancer and its effect on prognosis. *Br. J. Surg.*, **12**, 309.

FISHER, B., WOLMARK, N., ROCKETTE, H. & 14 others (1988). Postoperative adjuvant chemotherapy or radiation therapy for rectal cancer: results from NSABP protocol R-01. *J. Natl Cancer Inst.*, **80**, 21.

GASTROINTESTINAL TUMOR STUDY GROUP (1985). Prolongation of the disease-free interval in surgically treated rectal carcinoma. *New Engl. J. Med.*, **312**, 1465.

GEHAN, E.A. (1965). A generalized Wilcoxon test for comparing arbitrarily singly-censored data. *Biometrika*, **52**, 203.

JENSEN, H.E., NIELSEN, J. & BALSLEV, I. (1970). Carcinoma of the colon in old age. *Ann. Surg.*, **171**, 107.

KAPLAN, E.L. & MEIER, P. (1958). Non-parametric estimation from incomplete observations. *J. Am. Statist. Soc. C*, **53**, 457.

KRONBORG, O., FENGER, C., BERTHELSEN, K. & 11 others (1988). Escape clauses in a multicentre trial. Unforeseen problems and their potential influence on the general validity of the conclusions. *Theor. Surg.*, **2**, 157.

MANTEL, N. (1966). Evaluation of survival data and two new rank order statistics arising in its consideration. *Cancer Chemother. Rep.*, **50**, 163.

MAYER, R.J., O'CONNELL, M.J., TEPPER, J.E. & WOLMARK, N. (1989). Status of adjuvant therapy for colorectal cancer. *J. Natl Cancer Inst.*, **81**, 1359.

MOHIUDDIN, M., DERDEL, J., MARKS, G. & KRAMER, S. (1985). Results of adjuvant radiation therapy in cancer of the rectum. *Cancer*, **55**, 350.

QUIRKE, P., DIXON, M.F., DURDEY, P. & WILLIAMS. N.S. (1986). Local recurrence of rectal adenocarcinoma due to inadequate surgical resection. *Lancet*, **ii**, 996.

RICH, T.A., GUNDERSON, L.L., LEW, R., GALDIBINI, J.J., COHEN, A.M. & DONALDSON, G. (1983). Patterns of recurrence of rectal cancer after potentially curative surgery. *Cancer*, **52**, 1317.

SEEFELD, P.H. & BARGEN, J.A. (1943). The spread of carcinoma of the rectum: invasion of lymphatics, veins and nerves. *Ann. Surg.*, **118**, 76.

SUGARBAKER, P.H., GUNDERSON, L.L. & WITTES, R.E. (1985). Colorectal cancer. In *Cancer. Principles and Practice of Oncology*, DeVita, V.T., Hellman, S. & Rosenberg, S.A. (eds) p. 795. J.B. Lippincott Company: Philadelphia.

TANNOCK, I.F. (1989). Combined modality treatment with radiotherapy and chemotherapy. *Radiother. Oncol.*, **16**, 83.

TARONE, R.E. (1975). Tests for trend in life table analysis. *Biometrika*, **62**, 679.

TEPPER, J.E., COHEN, A.M., WOOD, W.C., ORLOW, E.L. & HEDBERG, S.E. (1987). Postoperative radiation therapy of rectal cancer. *Int. J. Radiat. Oncol. Biol. Phys.*, **13**, 5.

VIGLIOTTI, A., RICH, T.A., ROMSDAHL, M.M., WITHERS, H.R. & OSWALD, M.J. (1987). Postoperative adjuvant radiotherapy for adenocarcinoma of the rectum and rectosigmoid. *Int. J. Radiat. Oncol. Biol. Phys.*, **13**, 999.

WANEBO, H.J., RAO, B., PINSKY, C. & 4 others (1978). Preoperative carcinoembryonic antigen level as a prognostic indicator in colorectal cancer. *N. Engl. J. Med.*, **299**, 448.